# Identification of QTLs Conferring Resistance to Deltamethrin in *Culex pipiens pallens*

**DOI:** 10.1371/journal.pone.0140923

**Published:** 2015-10-20

**Authors:** Feifei Zou, Chen Chen, Daibin Zhong, Bo Shen, Donghui Zhang, Qin Guo, Weijie Wang, Jing Yu, Yuan Lv, Zhentao Lei, Kai Ma, Lei Ma, Changliang Zhu, Guiyun Yan

**Affiliations:** 1 Department of Pathogen Biology, Nanjing Medical University, Nanjing, Jiangsu, PR China; 2 Program in Public Health, University of California Irvine, Irvine, California, United States of America; Institute of Vegetables and Flowers, Chinese Academy of Agricultural Science, CHINA

## Abstract

*Culex pipiens pallens* is the most abundant Culex mosquito species in northern China and is an important vector of bancroftian filariasis and West Nile virus. Deltamethrin is an insecticide that is widely used for mosquito control, however resistance to this and other insecticides has become a major challenge in the control of vector-borne diseases that appear to be inherited quantitatively. Furthermore, the genetic basis of insecticide resistance remains poorly understood. In this study, quantitative trait loci (QTL) mapping of resistance to deltamethrin was conducted in F_2_ intercross segregation populations using bulked segregation analysis (BSA) and amplified fragment length polymorphism markers (AFLP) in *Culex pipiens pallens*. A genetic linkage map covering 381 cM was constructed and a total of seven QTL responsible for resistance to deltamethrin were detected by composite interval mapping (CIM), which explained 95% of the phenotypic variance. The major QTL in linkage group 2 accounted for 62% of the variance and is worthy of further study. 12 AFLP markers in the map were cloned and the genomic locations of these marker sequences were determined by applying the Basic Local Alignment Search Tool (BLAST) tool to the genome sequence of the closely related *Culex quinquefasciatus*. Our results suggest that resistance to deltamethrin is a quantitative trait under the control of a major QTL in *Culex pipiens pallens*. Cloning of related AFLP markers confirm the potential utility for anchoring the genetic map to the physical map. The results provide insight into the genetic architecture of the trait.

## Introduction

Mosquitoes transmit many damaging vector-borne diseases including malaria, dengue fever, yellow fever, encephalitis and filariasis [1]. According to the latest estimates of the World Health Organization (WHO) released in December 2013, there were about 207 million cases of malaria in 2012, 50–100 million dengue infections worldwide every year, and over 120 million filariasis cases [2]. *Culex pipiens pallens* is widely distributed across the globe, is one of the major disease-carrying insect vectors, and is largely responsible for periodic bancroftian filariasis and West Nile fever [3,4]. Deltamethrin (DM), an important synthetic pyrethroid insecticide, has made a valuable contribution to controlling mosquito populations. The insecticide binds to voltage-gated sodium channels in neuronal membranes, then causes nerve cells to produce repetitive discharges and eventually paralysis. However, continuous and extensive application of insecticides leads to the development of insecticide resistance, and has become the major obstacle to controlling the insect vector-borne diseases [5,6,7]. Determination of the mechanisms underpinning insecticide resistance will assist the development of much-needed novel strategies for managing insecticide resistance and disease control.

Resistance to insecticides is a complex genetic phenomenon that operates through polygenetic inheritance. There are two major causes of insecticide resistance; alterations in the insecticide target sites, and increased insecticide metabolism and excretion. Many genes are involved in resistance such as the sodium channel, cytochrome P450s, esterases and glutathione S-transferases [8,9,10,11]. However, the complexity of the resistance has not been fully elucidated.

Quantitative Trait Loci (QTL) refers to genome regions that influence quantitative characteristics, and mapping QTLs is the first step in the process of positional cloning and application of marker-assisted selection or introgression in genetic improvement [12]. The technology does not make any assumptions about resistance mechanisms, and can search for candidate loci throughout the whole genome but not to the level of individual genes. QTL mapping has been successfully used to study complex phenotypes including insecticide resistance. Ranson et al. found that two QTLs together explained over 50% of the variance in susceptibility to DDT in *Anopheles gambiae* [13]. Subsequently they identified genes involved in permethrin resistance that encoded cytochrome P450s and the sodium channel [14]. Other studies combined QTL mapping with positional cloning to identify P450 clusters genetically associated with pyrethroid resistance in *Anopheles funestus* and in *Aedes aegypti* [15,16,17,18]. But little is known regarding QTLs associated with insecticide resistance in *C*. *pipiens*. Previous studies have mainly focused on a single gene [19,20]. It is unknown whether resistance is the result of a single major gene cluster or if numerous gene families each contribute a small but additive effect. The mapping can solve the problem[21]. Further, it will aid our understanding of the genetic background that governs resistance, and will also help to facilitate positional cloning and the identification of related genes.

In this study, we used amplified fragment length polymorphism (AFLP) to construct a genetic map of *C*. *pipiens pallens* and used this to identify the QTL(s) responsible for DM resistance. The AFLP method produces dominant markers but does not need prior information for PCR primers, and provides fast and easy developed markers that can be positioned throughout the genome in any organism [22,23]. The genome of *Culex quinquefasciatus* (Southern house mosquito) has been sequenced and diverges little from *C*. *pipiens pallens* (Northern house mosquito); both belong to the *Culex pipiens* complex of mosquitoes (or incipient species—the taxonomy remains unclear) [24,25,26]. Mosquitoes have three chromosomes, and AFLP marker sequences have been successfully obtained by excision and sequencing of the target fragment [27,28,29]. Based on the known genome sequence location, AFLP markers of *C*. *pipiens pallens* were assigned to similar genomic regions (supercontigs) to the *C*. *quinquefasciatus* reference genome. These findings lay the foundation for future studies that combine linkage mapping and physical mapping of *C*. *pipiens pallens* and that use AFLP approaches more broadly.

In the present study, we developed the first AFLP-based genetic map of the reciprocal F_2_ intercross between susceptible and resistant strains of *C*. *pipiens pallens*. Based on the map and DM-resistance phenotypes, we detected seven QTLs affecting DM resistance and successfully obtained non-ambiguous sequences of 12 linked AFLP markers. A BLAST search confirmed that these markers occupied unique positions in the genome. These results will help to clarify the molecular mechanisms of insecticide resistance.

## Materials and Methods

### Mapping population, deltamethrin-resistance phenotype and DNA extraction

Two homozygous populations were used for reciprocal F_2_ intercross mapping populations. A DM-susceptible strain of *C*. *pipiens pallens* (the 50% lethal concentration LC_50_ = 0.008 mg/L) was obtained from Tangkou Village (Shandong Province), and has been colonized in the insectary without exposure to any insecticides since 2009 [30]. The DM-resistant strain was selected from its early fourth-instar larvae by selection with DM for about 40 generations to reach a 400-fold resistance (LC_50_ = 3.2 mg/L) following Li et al. [31]. A detailed selection procedure was described previously [32].

Two segregating populations were set up for QTL mapping. The first one (hereafter referred to as cross R♀-S♂) was established using pairwise mating between virgin resistant females and susceptible males; the second (cross S♀-R♂) was the result of mating between susceptible females and resistant males. In order to raise the ratio of fertilization between different strains, a male parent and 5–6 female parents were mated. Meanwhile we set a control population between susceptible females and males (cross S♀-S♂). The parents were allowed to mate for 3 days and the male was frozen at -80°C. Females were fasted for 3 hours and blood fed with live mice for 24 hours. Individual egg rafts were reared in separate rearing enamelwares, and pupae were transferred to cages for adult exclusion and sibling mating. For the F_1_ families, pupae were separated by sex, then one female and one male pupae from the same parents were allowed to emerge, mate and blood fed to obtain an F_2_ progeny. For F_2_ individuals, the larvae dipping method was used to test the resistance of larvae to DM in three populations (R♀-S♂, S♀-R♂, S♀-S♂). We got the concentration of deltamethrin caused about 50% mortality among the fourth-instar larvae as the method by Chen et al.[32]. The LC_50_ was calculated using the Probit analysis and Abbott's correction for mortality rate. All mosquito were reared and bred at 26±1°C, 80±5% RH and 16L: 8D light cycle. Adults were supplied with 5% sucrose solution and larvae were fed on yeast and powder of rabbit liver. A total of 279 F_2_ female mosquitoes were obtained from the cross R♀-S♂, while 208 F_2_ female individuals were from the cross S♀-R♂.

According to the WHO susceptibility test (2013), F_2_ female mosquitoes of cross R♀-S♂ and S♀-R♂ were exposed to 0.05% DM for 1 hour then transferred back to the holding tubes to recover and maintained with 5% sucrose solution for 24 h. The 1 hour recording of knock down are made at regular intervals, after 10, 20, 30, 40, 50 and 60 minutes into the exposure period. All parents, F_1_ progeny and F_2_ female progeny were collected and kept in silica gel-containing eppendorf tubes for DNA extraction and AFLP genotype determination. Knock-down time was analyzed by the Kaplan-Meier method using the Wilcoxon and a Log-rank method. Statistical analysis was performed using SPSS 13.0.

### BSA and AFLP analysis

A combination of Bulked Segregation Analysis (BSA) and AFLP analysis was used to identify DM resistance-associated molecular markers and genome regions. Resistant and susceptible bulks were prepared from two F_2_ groups by pooling equivalent amounts of total DNA from each of the 10 most resistant and the 10 most susceptible organisms. Mosquitoes still alive 24 h after DM exposure were chosen as the most resistant individuals, while those knocked down within 20 min and did not recover were deemed the most susceptible.

AFLP analysis was conducted on parents and two bulked pools as described by Vos et al.[33] with modifications. Briefly, restriction digestion with *Eco*RI and *Mse*I, adapter ligation and PCR amplification including pre-amplification and selective amplification were essential (S1 Table). A total of 136 primer combinations were screened, and PCR product polymorphism screens were conducted using an ABI 3730xl DNA Analyzer for high throughput capillary electrophoresis. Selective *Eco*RI primers were labeled with Liz500 (FAN), and 35–500 bp standards were used for size comparison. Allele sizes were analyzed using Gene Marker V1.80 (val) computer software (ABI) and marker data were exported in a matrix format.

Based on BSA-AFLP analysis, two essential criteria for selecting candidate primer combinations from the 136 primer pairs were established. Firstly, primer combinations must generate polymorphic fragments with a clear dominance inheritance pattern, showing dominance expression in one parent and complete recessive expression in the other. Secondly, the polymorphism between parents must be consistent with the polymorphism between two pools. A total of 29 primer pairs produced markers with clear dominant inheritance patterns and good reproducibility (S2 Table). All mosquitoes including parents, F_1_ and F_2_ individuals were genotyped with 29 primer pairs. In order to exam the *kdr* L1014 allele polymorphism in the mapping populations, the para-type sodium channel gene D2S6 region was amplified and sequenced as the description of Chen et al [32].

### Genetic linkage map construction

The Chi-squared test was used to determine if genotype met the Mendelian inheritance ratio. In two F_2_ intercrosses, after exclusion of highly distorted outliers (*P* < 0.001), eligible polymorphic fragments showing a 3:1 segregation ratio were used to construct the genetic linkage map. Linkage analysis and map construction were accomplished with the computer program JoinMap, version 4.0 (J.W. van Ooijen, 2006). AFLP fragments were converted to categories a, b, c and d according to the manual, then these markers were recorded in a TXT file and imported to the software. Linkage groups were formed based on four criteria: independence text LOD score, linkage LOD score, independence text *p*-value and recombination frequency in the software. In the R♀-S♂ cross, three linkage groups were determined by JoinMap with a LOD score of 6.0 and the relatively high LOD score reduced the rate of false positives to 10^−6^. The Haldane mapping function was used to convert recombination units into genetic distance for all linkage groups and the ripple value was 1.0. Then we reset the mapping function to Kosambi so as to verify the orders from Haldane method. Both methods should lead to more-or-less the same result.

### QTL mapping of deltamethrin resistance

The association between AFLP markers and resistance phenotype was analyzed by the QTL Cartographer 2.5 package. Using the WHO bioassay procedure, the survival time of resistant mosquitoes could not be quantified, so DM-resistance phenotypes were simplified into three categories (0, 1, 2). Individuals scored 0 were knocked down within the first hour and did not recover, individuals scored 1 were knocked down at some point, but recovered by 24 hours post exposure and individuals scored 2 were never knocked down over the testing period. The genetic map and phenotype data were imported into QTL Cartographer 2.5 (WinQTLCart2.5). In the Windows QTL Cartographer 2.5, composite interval mapping (CIM) tests whether an interval between two markers contains a QTL while simultaneously controlling for the effect of proximal QTLs located outside the interval. Model 6 was performed with a 10 cM window size, a value of 5 for control markers and a forward regression method. A 1000 permutation test at 95% confidence level and a walking speed of 1 cM to determine the LOD thresholds, with significance as 0.05 were set. The individual QTL designation was performed using the following format: DR-N, where DR = DM resistance, N = the digit to distinguish common QTLs.

### AFLP sequencing and location in the reference genome

A total of 15 AFLP loci placed on the linkage map were used to perform comparative mapping analysis with *C*. *quinquefasciatus*, as the flanking regions of AFLP markers have the ability to identify genomic regions that are homologous across species. PCR products were electrophoresed on 8% denaturing polyacrylamide gels using a BIO-RAD POWER-PAC 1000 and silver stained. Target AFLP bands were excised and eluted using an E.Z.N.A. Poly-Gel DNA Extraction Kit (Omegabiotek). Eluted products were reamplified by PCR using the same selective-amplification primers. The parameters for PCR were as the protocol of Vazyme Taq Plus DNA Polymerase. Reamplified products were electrophoresed on a 1.5% agarose gel, and appropriate bands were excised and further purified using the AxyPrep DNA Gel Extraction Kit. Purified DNA was cloned into the pMD-19T Simple Vector. Recombinant plasmid DNA was isolated and sequenced by the Beijing Genomic Institute. Sequences were accepted if identical from two different individuals.

Clean sequences of all 12 markers (L3A9.119, L1B1.151, L3A8.177, L2A5.138, L1B2.90, L1A16.146, L1A42.114, L4B1.175, L3A8.139, L1A16.95, L1A42.127, L1A16.134) were obtained and were searched against the *C*. *quinquefasciatus* reference genome using BLAST (https://www.vectorbase.org/blast). Markers were selected when sequences were assigned to a unique genomic position with an identity score of more than 90%.

## Results

### AFLP marker polymorphism

Among the 136 primer combinations tested, 54 pairs amplified bands that differed between parents. Based on BSA analysis, 29 pairs of primers that produced polymorphic fragments with clearly dominant inheritance and good reproducibility on F_2_ intercross populations were selected. The number of eligible fragments from the crosses R♀-S♂ and S♀-R♂ were 209 and 210, respectively. Of these, 102 (49.5%) were from the resistant strain and 108 (51.4%) were from the susceptible strain (S2 Table). On average, each primer combination produced 7.24 fragments and the fragment size ranged from 57 to 432 bp. The L2A5 primer combination produced the most fragments (13 in total, 6 from resistance individuals and 7 from susceptible individuals) while the L1A28 and L1A29 combinations produced the fewest fragments (three). A 521-bp fragment of the para-type sodium channel gene D2S6 region was amplified and sequenced; the results indicated that there was no polymorphism at the *kdr* allele, which showing L1014F mutation in the mapping parents, F_1_, and F_2_ populations.

### Phenotypic variability in resistance to DM

Resistance is commonly monitored by bioassay, by determining LC_50_ or by using uniform diagnostic doses[34]. In the F_2_ groups, the larvae dipping method was to detect the resistance to DM of larvae in cross R♀-S♂ and S♀-R♂, and cross S♀-S♂ was the control group with LC_50_ of about 0.01 mg/L (95% CI, 0.007–0.016). The resistance to DM was 29-fold higher in R♀-S♂ (LC_50_ = 0.29 mg/L; 95% CI, 0.22–0.39) compared with in control group, and was 10-fold higher in S♀-R♂ (LC_50_ = 0.10 mg/L; 95% CI, 0.06–0.16) compared with in the control. When the LC_50_ is measured for populations, usually a high resistance level is defined as a resistance ratio (LC_50_ of population divided by LC_50_ of susceptible strain) greater than 20, a moderate resistance level is defined as a resistance ratio between 10 and 20 and a low resistance level is defined as a resistance ratio between 2 and 10[34]. So in the two F_2_ intercross populations, the R♀-S♂ had a high resistance level to DM, while the S♀-R♂ had a low level.

**Fig 1:**
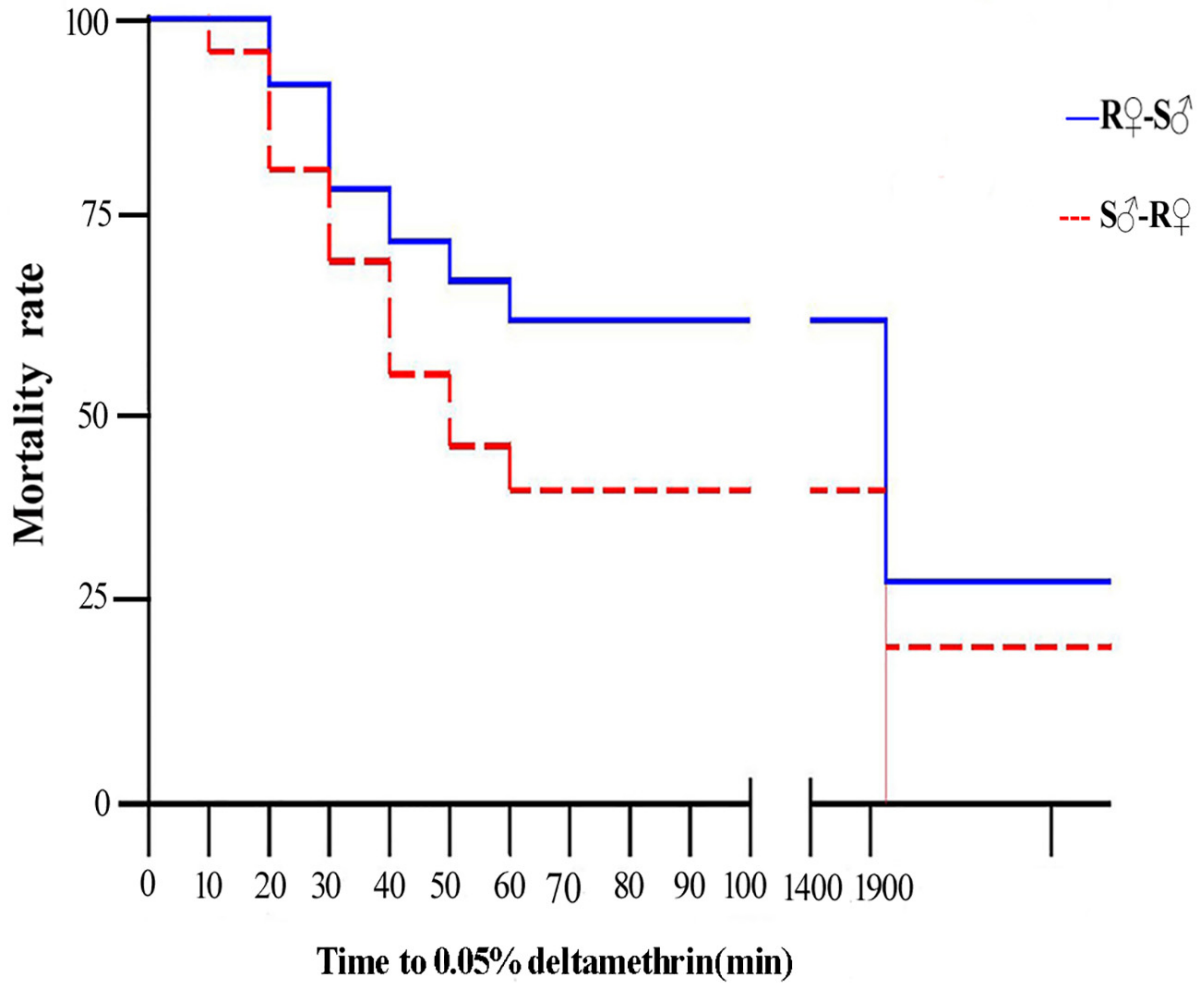
The difference in mortality rate between two *Culex pipiens pallens* crosses. The difference in mortality between the R♀-S♂ and S♀-R♂ crosses was significant at the *p* < 0.05 level using the Kaplan-Meier method.

A total of 279 F_2_ female individuals in the R♀-S♂ cross and 208 in the S♀-R♂ cross were obtained, respectively. The time taken for 50% of individuals to be knocked down (KDT50) was 61.784 min for the R♀-S♂ cross, which was significantly higher than that of the S♀-R♂ cross (45.682 min) as calculated by the Wilcoxon rank sum test (*p* < 0.05), and grouping was the major factor affecting the KDT50. The Kaplan-Meier method was also used to draw survival curves of two crosses (Fig 1), the results of which were in good agreement with the Wilcoxon rank sum test results (Chi-squared = 10.123, *p* < 0.05). So the significance level of the phenotypic difference between two cross groups could be detected by both LC_50_ and KDT50.

### AFLP-based map

**Fig 2:**
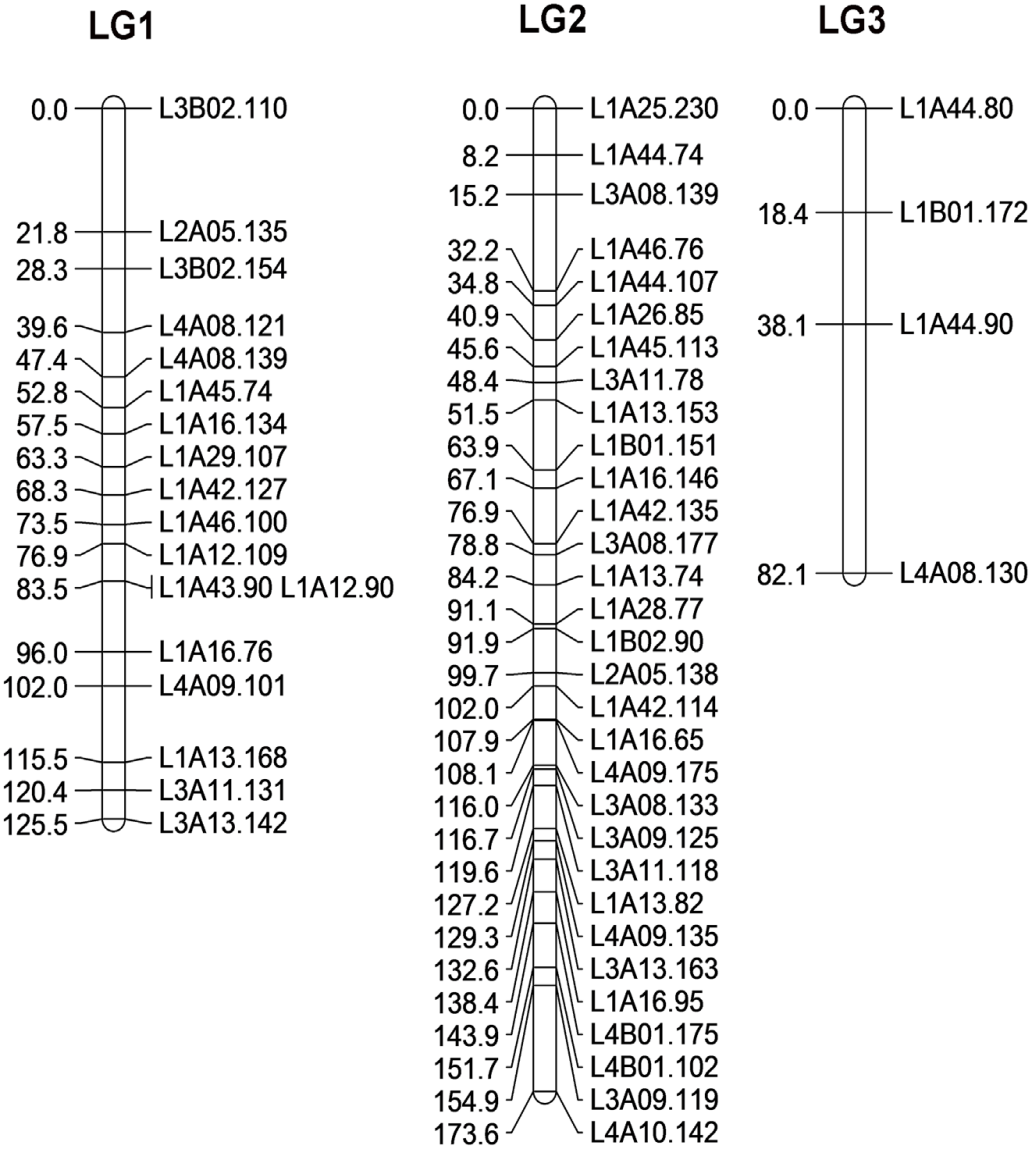
AFLP-based linkage map in *Culex pipiens pallens*. Three linkage groups are arranged at an LOD of 6.0 (*p* < 0.05). Numbers on the left side of each linkage group are genetic distances (cM) and AFLP markers are shown on the right.

Among 209 polymorphic fragments in the R♀-S♂ cross, 87 fragments fitted a 3:1 segregation ratio (*p* > 0.001), while in the S♀-R♂ cross, 80 fragments were detected. These markers were used to construct a linkage map separately. In the R♀-S♂ cross, 53 markers were assigned to three linkage groups (LGs) at an LOD threshold of 6.0 (*p* < 0.05). The other markers could not be assigned to any group. The final map covered a total length of 381 cM. Lengths of three LGs were 125.5, 173.6 and 82.1 cM, and the numbers of markers in three LGs were 18, 31 and 4, respectively. The average spacing size in the distance between two markers was 7 cM and the largest distance between two markers was 44 cM in LG 3. The linkage order and position of these markers was determined in Fig 2. However, we constructed the linkage map of cross S♀-R♂ with the LOD from 1.0 to 10.0 and LOD incremented of 1.0. But the loci were distributed randomly. We could not recover a linkage map with only 3 groups. Therefore only the R♀-S♂ cross was further analyzed.

### QTL analysis

**Fig 3:**
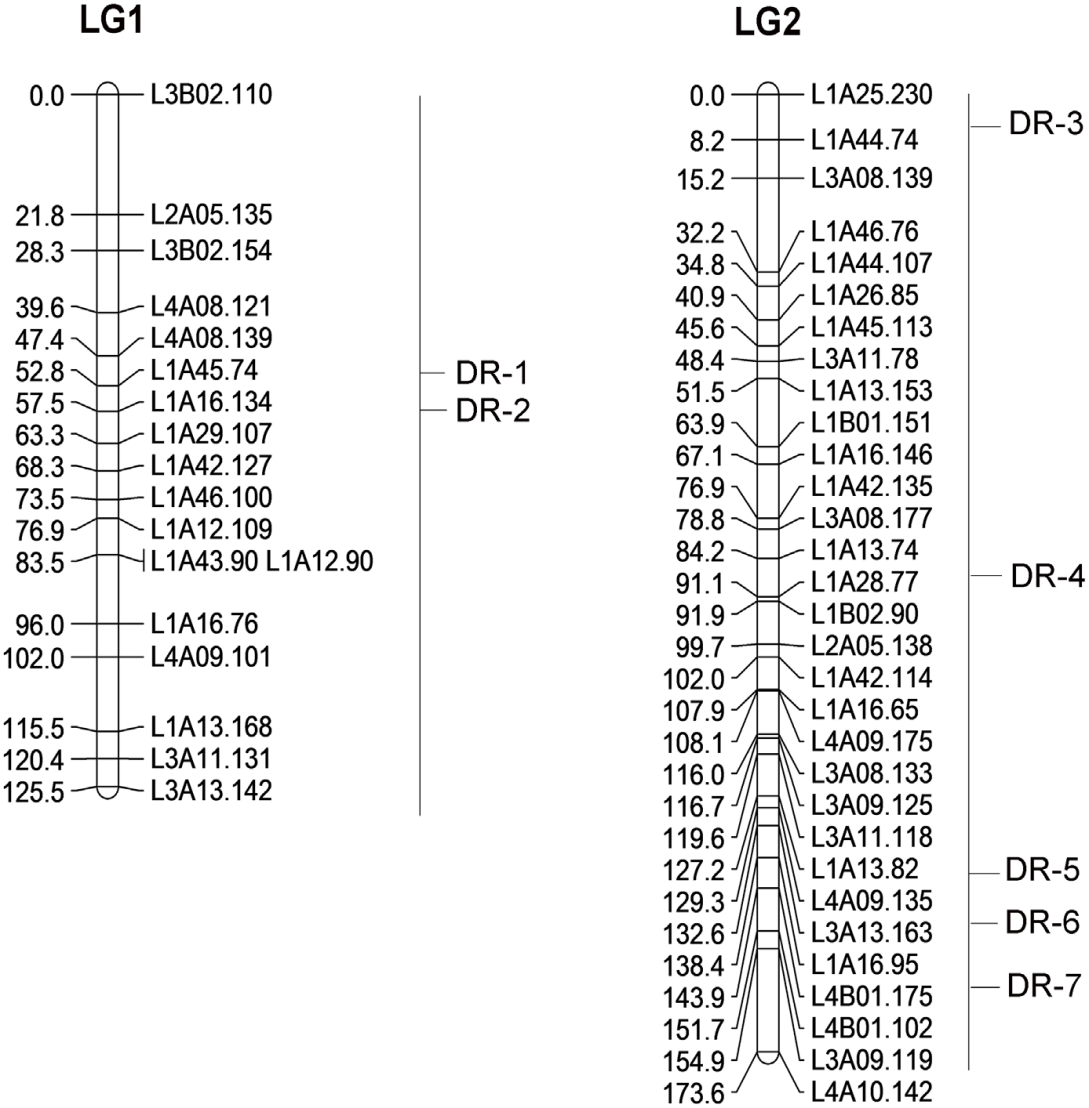
Putative QTLs for deltamethrin resistance in *Culex pipiens pallens*. The position of QTLs identified with composite interval mapping (CIM) are indicated as DR-1~DR-7 from LG1 and LG2.

The association between AFLP markers and resistance was examined in the R♀-S♂ cross with 279 individuals. QTL analysis was performed using CIM algorithm as described above. Seven QTLs were identified, namely DR-1, DR-2, DR-3, DR-4, DR-5, DR-6 and DR-7 (Fig 3).

Using the CIM method, it is more powerful than other QTL mapping procedures as the effects of other QTLs are not treated as residual variance. Furthermore, it can reduce the bias that would normally be associated with a QTL that is linked to the position being tested [35,36]. All statistical parameters were obtained by CIM (Table 1). The LOD score plots for linkage groups with identified QTL provide a basis for identifying molecular markers most closely linked to the QTL (S1 Fig). The permutation test indicated that all QTLs were statistically significant (*p* < 0.05; LOD > 3.0). The LOD score peak of each of these QTLs exceeded 3.0 and were from 3.58 to 9.57. The nearest markers of DR-1, DR-2, DR-3, DR-4, DR-5, DR-6 and DR-7 were L4A8.139, L1A16.134, L1A44.74, L1A28.77, L4B1.175, L4B1.175, L4A10.142, respectively. These QTLs accounted for approximately 95% of the total phenotypic variation and the contribution of each QTL ranged from 0.98% to 61.53%. Notably, DR-6 between L4B1.175 and L4B1.102 in linkage group 2 made by far the biggest contribution with a LOD of 6.15 and the highest additive effect (0.95), which was the major QTL for the resistance. The DR-4 associated with marker L1A13.74 and L1A28.77 had the highest LOD score (9.57) and explained 15.15% of the phenotypic variation. The additive effects of these QTLs ranged from –0.54 to 0.95, and their dominance effects ranged from -0.46 to 0.58.

**Table 1:** QTL positions and genetic effects associated with deltamethrin resistance in *Culex pipiens pallens*. Marker intervals for QTL positions are in parentheses next to composite interval mapping intervals from Fig 3. LG = linkage group, A = additive effect, D = dominant effect, R^2^ = proportion of the QTL accounting for the phenotypic variation in susceptibility to deltamethrin in *Culex pipiens pallens*.

QTL	LG	Marker interval (cM)	95% CI (cM)	LOD (cM)	A	D	R^2^ (%)
DR-1	1	L4A8.139-L1A45.74	47.9–51.2	4.8 (49.5)	-0.38	0.31	2.38
DR-2	1	L1A45.74-L1A16.134	54.1–61.1	5.7 (56.9)	-0.54	0.58	5.67
DR-3	2	L1A25.230-L1A44.74	5.6–7.1	3.6 (6.2)	-0.39	0.36	1.53
DR-4	2	L1A13.74-L1A28.77	87.0–89.7	9.6 (88.4)	-0.06	0.07	15.15
DR-5	2	L1A16.95-L4B1.175	141.7–142.8	3.8 (142.6)	0.23	-0.46	0.98
DR-6	2	L4B1.175-L4B1.102	144.8–147.0	6.2 (146.2)	0.95	-0.21	61.53
DR-7	2	L3A9.119-L4A10.142	163.7–169.8	8.6 (165.2)	-0.12	-0.24	7.80

### Comparative mapping

Comparative genomics can provide valuable information about the architecture and functional organization of a species’ genome. Full-length sequences of 12 selected markers were obtained at least from two different individuals (S4 File). These sequences were searched against the *C*. *quinquefasciatus* genome sequence using BLAST tool, and all were located on different supercontigs in regions of high sequence identity (>90%; Table 2). Based on the analysis described by Arensburger et al. (2010), marker L1B1.151 in supercontig 3.67 was mapped to a location on Chromosome 2, and the unique correspondences of LG2 and Chromosome 2 was confirmed.

**Table 2:** Description and genomic location of marker sequences in *Culex pipiens pallens*.

Marker	Sequence length (bp)	Location in the *Culex quinquefasciatus* genome(supercontig)	Identity (%); e-value
L3A9.119	118	3.289: 417301–417393	97.9; 1e-37
L1B1.151	154	3.67: 123187–123314	94.6; 7e-49
L3A8.177	181	3.388: 393117–393273	99.3; 9e-74
L2A5.138	137	3.98: 137570–137681	99.1; 2e-49
L1B2.90	92	3.21: 109702–109770	95.7; 1e-23
L1A16.146	145	3.45: 233279–233399	99.2; 2e-54
L1A42.114	113	3.20: 110586–110677	97.0; 5e-36
L4B1.175	184	3.492: 253145–253304	97.5; 1e-71
L3A8.139	134	3.453:169422–169449; 3.453: 169467–169553	92.8; 0.00889.1; 2e-22
L1A16.95	99	3.560:24436–24498	92.1; 1e-17
L1A42.127	128	3.1618:14612–14714	100; 3e-46
L1A16.134	132	3.174:423860–423967	95.4; 8e-42

## Discussion

### QTL analysis of deltamethrin resistance

In this study, seven QTLs that affected resistance to DM in *C*. *pipien pallens* were identified by CIM method. Multiple QTL regions confirmed that DM resistance is a quantitative trait in this organism, and the identified QTLs accounted for 95% of the phenotypic variance. DR-7 was the major QTL and accounted for approximately 61% of the variance, while the other six QTLs contributed 34% between them. These results are in agreement with a previous study which identified three QTLs accounting for 97.4% of the pyrethroid resistance in *Anopheles funestus*, and one QTL accounted for 96% of the variance in this case [16]. The different number of QTLs detected can be explained by species differences, the markers used, the mapping population size, and individual gene effects [37]. A relatively large sample size of 279 F_2_ individuals was used for QTL analysis, and some minor QTLs could be detected as a result.

There were so many genes involved in insecticide resistance in *Culex* mosquitoes, *kdr*, cytochrome P450s, GST, ESTs. Candidate gene approaches investigated the molecular mechanisms of insecticide resistance were limited in scope due to a focus upon one to few loci and could not take into the account of the multiple and polygenic modes in *Culex* mosquitoes. In the study, both parents and mapping population were detected with pure L1014F mutation of the para-type sodium channel gene, suggesting the metabolic gene responsible for the deltamethrin resistance. Recently, the development of next-generation sequencing (NGS) technologies provides an ideal method for rapid and reliable genomic exploration of mosquitoes. But all of these arrays could not determine the genetic role for specific loci in DM resistance and the proportion of loci accounting for the phenotypic variation. The major finding of this study is that resistance to deltamethrin is under the control of one major QTL, but several QTLs play more minor roles in *C*. *pipien pallens*. Most of the identified QTLs were located in linkage group 2, suggesting an significant role of chromosomes 2 in insecticide resistance. These will be responsible for the molecular mechanisms of insecticide resistance. While a lack of a high-quality chromosome-based genome assembly for *Culex* mosquito remained a significant impediment to clone the major QTL interval. For the major QTL, advanced intercross lines will be established in the future to identify additional genetic markers and better define the interval in order to isolate the major genes responsible for DM resistance.

### Genetic linkage map of *C*. *pipiens pallens*

A linkage map is a genetic tool that can be used to study genome structure and organization, and can display the relative positions of known genes and/or molecular markers. For the *Culex* genera of mosquitoes, several linkage maps have been reported [3,38,39,40] and for *Aedes aegypti*, high-resolution linkage maps were constructed using RFLP, SNP, SSCP, STS, and ESTs markers [41,42,43]. For *Anopheles* mosquitoes, complicated maps were constructed [13,14,44,45]. In *Aedes* and *Anopheles*, physical maps and linkage maps were well integrated based on known genome sequences [45,46]. In contrast, genetic studies on *Culex* mosquitoes are relatively fewer in number, and no relate QTL study was recorded, little is known about the genetic basis of DM resistance in *C*. *pipiens pallens*, the most prevalent mosquito species in northern China [32]. Here we first report the inchoate linkage map based on BSA-AFLP markers in a large segregating population, which was essential for subsequent QTL analysis. The final map had a total length of 381 cM and an average marker resolution of 7 cM. It has been shown that the ability to detect QTLs is nearly the same for a marker spacing of 10 cM, and only slightly decreases for a marker spacing of 20 or even 50 cM using linkage analysis when scoring an infinite number of marker. Even with an infinite number of markers, the confidence interval for QTL is strongly affected by population size and gene effect [37]. The map of 279 individuals was therefore deemed suitable for QTL mapping. Consistent with other studies, 6, 25 and 22 markers conferring permethrin resistance in *Anopheles funestus* were identified on chromosomes X, 2 and 3 of the linkage map [47], whereas only two markers were identified on the X chromosome and 26 and 47 markers were located on chromosomes 2 and chromosome 3 in *Anopheles gambiae* [48]. Zhong et al. (2006) also reported that AFLP markers provided good coverage [49]. The map with 18, 31 and 4 markers in the LG1, LG2 and LG3 generated in this study suggested that there was a considerable reduction in recombination in linkage group 3 compared with linkage groups 1 and 2. This finding is in agreement with earlier results that found a similar situation in other *Culex pipiens* complex mosquitoes [40].

Comparative mapping between *C*. *pipiens pallens* and *C*. *quinquefasciatus* give us new insights into the evolution of mosquito species. Given the sequence and location of marker L1B1.151 on chromosome 2, the combination of linkage group 2 and chromosome 2 could be detected. However, additional markers will be needed to improve the average marker resolution. Since 10 AFLP markers (L3A9.119, L1B1.151, L3A8.177, L2A5.138, L1B2.90, L1A16.95, L1A16.146, L1A42.114, L4B1.175 and L3A8.139) were mapped on the same linkage group (chromosome 2), we could anticipate that all of these supercontigs (3.289, 3.67, 3.388, 3.98, 3.21, 3.560, 3.45, 3.20, 3.492, 3.453) could be mapped to chromosome 2 [24]. This will form the basis of future to fill the gaps in the current genome assembly using a combination of linkage mapping and physical mapping.

## Conclusion

In summary, a genetic linkage map of *C*. *pipiens pallens* was constructed using BSA-AFLP analysis. The linkage map consists of 53 AFLP markers with a total length of 381 cM across 3 linkage groups. Seven QTLs influencing resistance to DM were detected in an F_2_ segregation population of 279 individuals. These QTLs accounted for 95% of the phenotypic variance of DM resistance, and one major QTL on linkage group 2 contributed 61% of the variance. Additionally, 12 AFLP markers were sequenced and located using the *C*. *quinquefasciatus* reference genome. All 12 located to unique supercontigs and 10 markers were mapped to chromosome 2. These results lay the foundation for future work to combine physical mapping with linkage mapping. Resistance to DM appears to be controlled by one major QTL and several minor QTLs, and further study is needed to identify the genes in the major QTL region.

## Supporting Information

S1 Table Adapter sequences, preselective and selective primer sequences of the AFLP markers. (DOC)

S2 Table Data of AFLP-based genotype and deltamethrin-resistance phenotype in total 503 mosquito from two crosses. (XLSX)

S1 Fig Composite interval mapping of deltamethrin resistance. Solid lines represent LOD estimated by composite-interval mapping in the Windows QTL Cartographer 2.5. Significance thresholds are indicated by horizontal lines, with LOD = 3.0 (*p* < 0.05) as determined by 1000 permutations of the mapping data.
(TIF)

S1 Text Full-length sequences of the 12 identified AFLP markers. (DOCX)
