# Supplementary material for: Identification of QTLs Conferring Resistance to Deltamethrin in Culex pipiens pallens
Source: PLoS One. 2015 Oct 20;10(10):e0140923. doi: 10.1371/journal.pone.0140923 (PMC4617896; doi:10.1371/journal.pone.0140923)
Supplement: S1 Table — (DOC) [file pone.0140923.s001.doc]

**S**1. Adapter sequences, preselective and selective primer sequences of the AFLP markers

|  | **Sequences** |
| --- | --- |
| **Adapter** |  |
| *Eco*RIoligo1 | GAC-TGC-GTA-CCA-ATT-CAC-A |
| *Eco*RIoligo2 | CTC-GTA-GAC-TGC-GTA-CC |
| *Mse*I oligo1 | GAC-GAT-GAG-TCC-TGA-G |
| *Mse*I oligo2 | TAC-TCA-GGA-CTC-AT |
| **Preselective primers (5’-3’)** |  |
| PRESEL-A | GAC-TGC-GTA-CCA-ATT-C |
| PRESEL-B | GAT-GAG-TCC-TGA-GTA-A |
| **Selective primers (5’-3’)**  ***Eco*RI primers** |  |
| L1 | GAC-TGC-GTA-CCA-ATT-CAC-A  5' Labelled FAM |
| L2 | GAC-TGC-GTA-CCA-ATT-CAA-C  5' Labelled FAM |
| L3 | GAC-TGC-GTA-CCA-ATT-CAA-G  5' Labelled FAM |
| L4 | GAC-TGC-GTA-CCA-ATT-CAC-C  5' Labelled FAM |
| ***Mse*I primers** |  |
| B1 | GAT-GAG-TCC-TGA-GTA-ACAC |
| B2 | GAT-GAG-TCC-TGA-GTA-ACAT |
| A1 | GAT-GAG-TCC-TGA-GTA-ACAA |
| A2 | GAT-GAG-TCC-TGA-GTA-ACAG |
| A3 | GAT-GAG-TCC-TGA-GTA-ACTC |
| A4 | GAT-GAG-TCC-TGA-GTA-ACTA |
| A5 | GAT-GAG-TCC-TGA-GTA-ACTT |
| A6 | GAT-GAG-TCC-TGA-GTA-ACTG |
| A7 | GAT-GAG-TCC-TGA-GTA-ACGC |
| A8 | GAT-GAG-TCC-TGA-GTA-ACGA |
| A9 | GAT-GAG-TCC-TGA-GTA-ACGT |
| A10 | GAT-GAG-TCC-TGA-GTA-ACGG |
| A11 | GAT-GAG-TCC-TGA-GTA-ACCC |
| A12 | GAT-GAG-TCC-TGA-GTA-ACCA |
| A13 | GAT-GAG-TCC-TGA-GTA-ACCT |
| A14 | GAT-GAG-TCC-TGA-GTA-ACCG |
| A15 | GAT-GAG-TCC-TGA-GTA-ATAC |
| A16 | GAT-GAG-TCC-TGA-GTA-ATAA |
| A17 | GAT-GAG-TCC-TGA-GTA-ATAT |
| A18 | GAT-GAG-TCC-TGA-GTA-ATAG |
| A19 | GAT-GAG-TCC-TGA-GTA-ATTC |
| A20 | GAT-GAG-TCC-TGA-GTA-ATTA |
| A21 | GAT-GAG-TCC-TGA-GTA-ATTT |
| A22 | GAT-GAG-TCC-TGA-GTA-ATTG |
| A23 | GAT-GAG-TCC-TGA-GTA-ATGC |
| A24 | GAT-GAG-TCC-TGA-GTA-ATGA |
| A25 | GAT-GAG-TCC-TGA-GTA-ATGT |
| A26 | GAT-GAG-TCC-TGA-GTA-ATGG |
| A27 | GAT-GAG-TCC-TGA-GTA-ATCC |
| A28 | GAT-GAG-TCC-TGA-GTA-ATCA |
| A29 | GAT-GAG-TCC-TGA-GTA-ATCT |
| A30 | GAT-GAG-TCC-TGA-GTA-ATCG |
| A31 | GAT-GAG-TCC-TGA-GTA-ACA |
| A32 | GAT-GAG-TCC-TGA-GTA-ACT |
| A33 | GAT-GAG-TCC-TGA-GTA-ACG |
| A34 | GAT-GAG-TCC-TGA-GTA-ACC |
| A35 | GAT-GAG-TCC-TGA-GTA-ATA |
| A36 | GAT-GAG-TCC-TGA-GTA-ATT |
| A37 | GAT-GAG-TCC-TGA-GTA-ATG |
| A38 | GAT-GAG-TCC-TGA-GTA-ATC |
| A39 | GAT-GAG-TCC-TGA-GTA-AAA |
| A40 | GAT-GAG-TCC-TGA-GTA-AAT |
| A41 | GAT-GAG-TCC-TGA-GTA-AAG |
| A42 | GAT-GAG-TCC-TGA-GTA-AAC |
| A43 | GAT-GAG-TCC-TGA-GTA-AGA |
| A44 | GAT-GAG-TCC-TGA-GTA-AGT |
| A45 | GAT-GAG-TCC-TGA-GTA-AGC |
| A46 | GAT-GAG-TCC-TGA-GTA-AGC |
| A47 | GAT-GAG-TCC-TGA-GTA-AA |
| A48 | GAT-GAG-TCC-TGA-GTA-AT |
| A49 | GAT-GAG-TCC-TGA-GTA-AG |
| A50 | GAT-GAG-TCC-TGA-GTA-AC |
