# Supplementary figures and images for: Identification of QTLs Conferring Resistance to Deltamethrin in Culex pipiens pallens
Source: PLoS One. 2015 Oct 20;10(10):e0140923. doi: 10.1371/journal.pone.0140923 (PMC4617896; doi:10.1371/journal.pone.0140923)

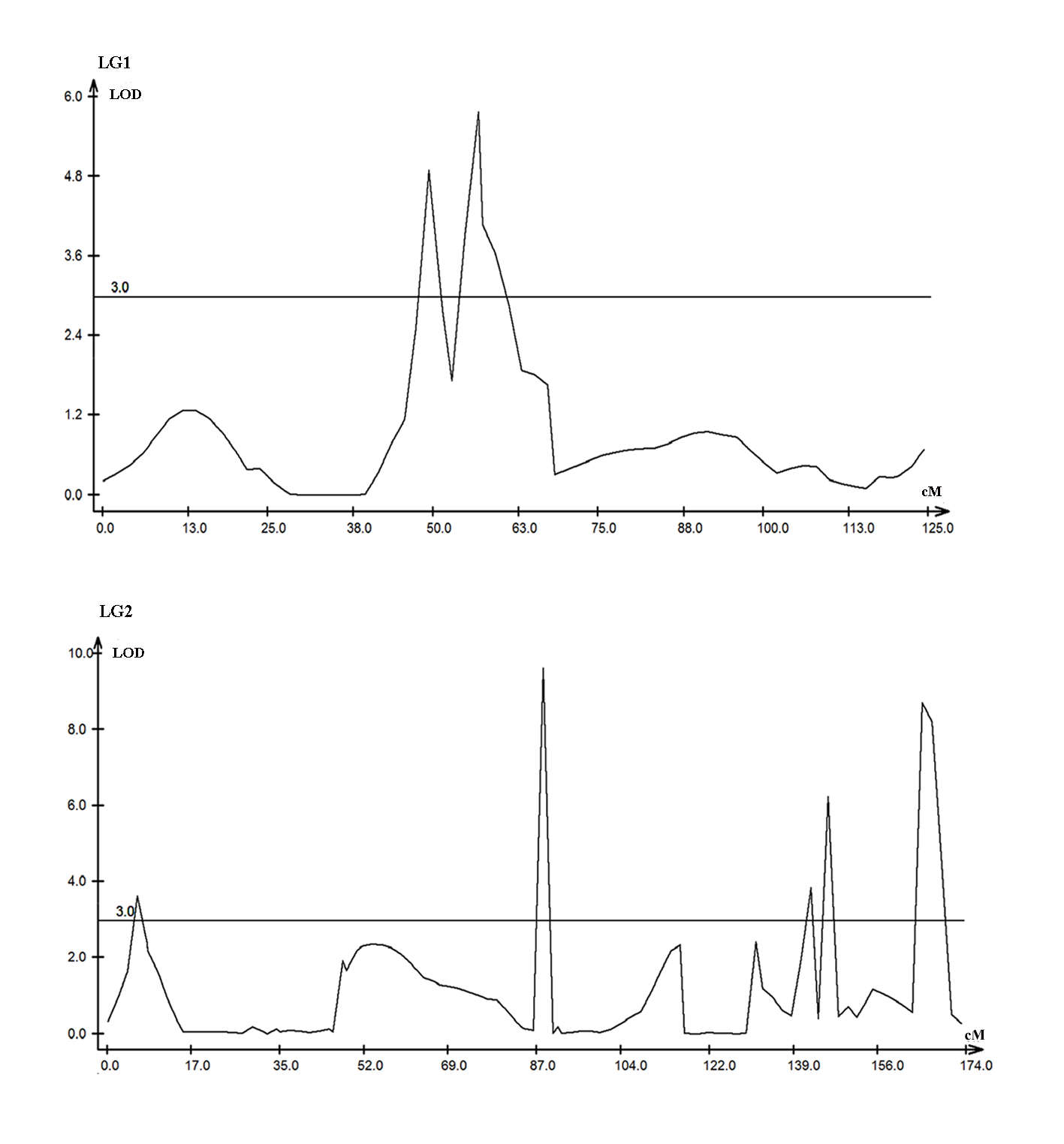

Supplement: S1 Fig — Solid lines represent LOD estimated by composite-interval mapping in the Windows QTL Cartographer 2.5. Significance thresholds are indicated by horizontal lines, with LOD = 3.0 (p < 0.05) as determined by 1000 permutations of the mapping data. (TIF) [file pone.0140923.s003.tif]
