# Supplementary material for: Identification of QTLs Conferring Resistance to Deltamethrin in Culex pipiens pallens
Source: PLoS One. 2015 Oct 20;10(10):e0140923. doi: 10.1371/journal.pone.0140923 (PMC4617896; doi:10.1371/journal.pone.0140923)
Supplement: S1 Text — (DOCX) [file pone.0140923.s004.docx]

**S1 Text. Full-length sequences of the 12 identified AFLP marker****s**

L1A16.95

GACTGCGTACCAATTCACAAAATAGGGTATATGTCCCTATTTTGGGCCTACTAAGTAAAGCTTCAAAACATTTTGCCTTGTTATTACTCAGGACTCATC

L1A16.134

GACTGCGTACCAATTCACATTTGCAGCACTCTGCCTAAAGCTCGCGTTCAACGGAGTCTCGGTGCAACATGGAATTGAACTTCGGCGAAGCGTAAACAAACAATGCGTGTGTGTTATTACTCAGGACTCATC

L1A16.146

GACTGCGTACCAATTCACACCTTTGGATTGATGGTCTGGGACGCTAAGCAGTCGGCCATCAGAAGGTTTACACTCTAGCAGTGAATTGATCCGTAGTGTTGAAACATGTTATCTCTTCTTCTCATATTATTACTCAGGACTCATC

L1A42.114

GACTGCGTACCAATTCACATAACCTCAACTGGTCGGGTCCGATCGCCACGAAAAAGCCATGTAGAGGGATGCCATTTTCCTCAAAGGCCGTGTCTGTTTACTCAGGACTCATC

L1A42.127

GACTGCGTACCAATTCACATGAGCAACTCTACAAATCACTGCACATTGACTTACAAATCAGCCCCGGAAGAGTTCTCCAAGCAGGAAGCGGTCGCCAGCAAAAGTGACGGTTTACTCAGGACTCATC

L1B1.151

GACTGCGTACCAATTCACACAAATGTGATCAACAACTCGCAAACTTTGACGAAACCTTGCAGACAGCTTGACGAGGTGTGAACGATCTCTCTCTGGGGGGGTCCATCGTAATTCATGTAAACCGAGAACTGCACCGTGTTACTCAGGACTCATC

L2A5.138

GACTGCGTACCAATTCAACTGGGTTCTAGGTAGAATGAGATGCTGCATAATTCTTTGATATCTGCCAAAGTTTATATTTGCCAATTTTATCCATTAGCAAACTTACGTCAAAAGCTGGAAGTTACTCAGGACTCATC

L1B2.90 GACTGCGTACCAATTCACAGAAGTAAAAAAAGCCTCCAAATTATTTACCGCTTTGTAGTCTGTGCAGTTGTATCATGTTACTCAGGACTCATC

L3A8.139

GACTGCGTACCAATTCAAGTCCCGCTGGCATTTTTCCACATATCCACCGTCAGTCAGTTTCGCAGAGAGGGGTGAAAGCTGAAGAGGAAGAAGTGAAATTTTCCCGAGGTTTTTCTCGTTACTCAGGACTCATC

L3A8.177

GACTGCGTACCAATTCAAGAAATTGAAGCAGAAGTATACAACGTCCTTTCTTTATGGCCCATTTTTTTTGAAAATTTACAAATAAAATAAAAATAGAGCCTACCTTGATTTCAATCTACTGCTCATATTGAAAAAAATCAGGTTTATTTCAGAAAATATTCATCGTTACTCAGGACTCATC

L3A9.119

GACTGCGTACCAATTCAAGAATTTCAGAGTTTCAGAATTTTAGTTTTGACAAAATTACATCAATTTTTTTTGTTCATATAAGAATACAAATTGGTAGCAACGTTACTCAGGACTCATC

L4B1.175

GACTGCGTACCAATTCACCTCGAGACAGCGACTTGTTCACGATATTCAGCAGAACTACTGCAATTACGTCCCAAGCATCCATAAGAACTCGATTCGTGACGACTTCCGTAGCCCTTCTTGTGGAAGGCTCCCAACAGCTCGTTTAGGTTACCCGTGGGCATGTTCTCGTTACTCAGGACTCATC
